# Synergistic Antimyeloma Activity of Dendritic Cells and Pomalidomide in a Murine Myeloma Model

**DOI:** 10.3389/fimmu.2018.01798

**Published:** 2018-08-03

**Authors:** Manh-Cuong Vo, Seoyun Yang, Sung-Hoon Jung, Tan-Huy Chu, Hyun-Ju Lee, Thangaraj Jaya Lakshmi, Hye-Seong Park, Hyeoung-Joon Kim, Je-Jung Lee

**Affiliations:** ^1^Research Center for Cancer Immunotherapy, Chonnam National University Hwasun Hospital, Hwasun, South Korea; ^2^Department of Hematology-Oncology, Chonnam National University Hwasun Hospital, Hwasun, South Korea; ^3^Vaxcell-Bio Therapeutics, Hwasun, South Korea

**Keywords:** dendritic cell, immunotherapy, pomalidomide, myeloma, cancer vaccine

## Abstract

We have previously shown that immunization with tumor antigen-loaded dendritic cells (DCs) and the immunomodulating drug, lenalidomide, synergistically potentiates the enhancing antitumor immunity in a myeloma mouse model. In this study, we investigated the immunogenicity of DCs combined with pomalidomide and dexamethasone in a myeloma mouse model. MOPC-315 cells were injected subcutaneously to establish myeloma-bearing mice. Four test groups were used to mimic clinical protocol: (1) PBS control, (2) DCs, (3) pomalidomide + dexamethasone, and (4) DCs + pomalidomide + dexamethasone. The combination of DCs plus pomalidomide and dexamethasone displayed greater inhibition of tumor growth compared to the other groups. This effect was closely related with reduced numbers of immune suppressor cells including myeloid-derived suppressor cells, M2 macrophages, and regulatory T cells, with the induction of immune effector cells such as CD4^+^ and CD8^+^ T cells, memory T cells, natural killer (NK) cells, and M1 macrophages, and with the activation of T lymphocytes and NK cells in the spleen. Moreover, the level of the immunosuppressive factor vascular endothelial growth factor was significantly reduced in the tumor microenvironment. The collective findings in the murine myeloma model suggest that tumor antigen-loaded DCs combined with pomalidomide and dexamethasone synergistically enhance antitumor immunity by skewing the immune-suppressive status toward an immune-supportive status.

## Introduction

Multiple myeloma (MM) is a clonal B-cell malignancy characterized by the aberrant accumulation of plasma cells within bone marrow (BM) and extramedullary site (1). Patients with MM suffer from renal failure, anemia, infection, hypercalcemia, osteolytic bone lesions, and immunodeficiency (2). Although the use of novel antimyeloma drugs such as immune modulatory drugs (thalidomide, lenalidomide, and pomalidomide) and proteasome inhibitors (bortezomib and carfilzomib) has remarkably improved the disease outcome and extended the overall survival, most patients with MM eventually relapse and develop resistance to their treatment (3, 4). Thus, new therapies are clearly needed.

Dendritic cells (DCs) may serve as potent antigen-presenting cells for antitumor immunity owing to their ability to very efficiently take up, process, and present antigens and play a pivotal role in initiating T cell-mediated immune response (5–13). In previous studies, we established several treatment platforms in murine tumor models to enhance the antitumor effects of vaccines comprising DCs combined with cyclophosphamide (14), toll-like receptor agonists (15), and the immunomodulatory drug (IMiD) lenalidomide (16). All these combined strategies focus on modulating the tumor microenvironments to augment antitumor activities of DC vaccines. In a previous phase I clinical trial, we demonstrated the feasibility and safety of DC vaccination when combined with cyclophosphamide in patients with relapsed or refractory MM (17). This study was undertaken to develop more potent treatment protocols for enhancing the response of a DC-based vaccine by overcoming the immunosuppressive microenvironment in patients with MM.

Pomalidomide is a strong IMiD that is an analog of thalidomide and lenalidomide to exhibit a potent antimyeloma effect (18). This antimyeloma effect of pomalidomide has been reported, including induction of antiproliferative and proapoptotic effects on MM cells (19, 20), and modulation of BM microenvironment to inhibit the binding of MM cells to BM stromal cells that mediate the production of growth factors and angiogenic molecules, including vascular endothelial growth factor (VEGF) (21). In addition, pomalidomide improves cellular immunity *via* the activation of immune effector cells, such as DCs, natural killer (NK) cells, and T cells (22) and the reduction of immune inhibitory cells, such as regulatory T cells, on tumor microenvironment (23, 24). Importantly, pomalidomide plus dexamethasone has synergistic antiproliferative effects in lenalidomide-resistant myeloma cells (25, 26). The activity of pomalidomide in cells that are resistant or refractory to lenalidomide may be due to important differences in both the potency of the drugs and their respective mechanisms of action (27–29). In clinical studies, pomalidomide displayed synergistic effects when combined with low-dose dexamethasone in patients with advanced MM who had been treated with lenalidomide and/or bortezomib (30–33).

In this study, we investigated whether a DC-based vaccine combined with pomalidomide and dexamethasone had a synergistic effect in a myeloma mouse model. This study closely mimicked a clinical MM treatment protocol and demonstrated that tumor antigen-loaded DC vaccination with pomalidomide and dexamethasone enhanced antitumor immunity in the mouse model by inhibiting immune-suppressive cells and by effector cell recovery and demonstrated superior polarization of the Th1/regulatory balance in favor of the Th1 immune response. Our study provides a preclinical rationale for understanding the role of DC vaccination when combined with pomalidomide and dexamethasone to inhibit tumor cell growth and restore immune function in myeloma-bearing mice.

## Materials and Methods

### Mice and Tumor Cell Lines

Six- to eight-week-old female BALB/c (H-2^d^) mice were purchased from Orient Bio (Iksan, Republic of Korea) and were maintained under specific pathogen-free conditions. All animal care, experiments, and euthanasia were performed in accordance with protocols approved by the Chonnam National University Animal Research Committee. The MOPC-315 murine plasmacytoma cell line (induced with mineral oil in a BALB/c mouse) and YAC-1 cell line were purchased from the American Type Culture Collection (Rockville, MD, USA). The cell lines were maintained in Dulbecco’s modified Eagle’s medium (DMEM; Gibco-BRL, Grand Island, NY, USA) supplemented with 10% (v/v) fetal bovine serum (FBS; Gibco-BRL) and 1% (w/v) penicillin/streptomycin (PS).

### IMiD (Pomalidomide)

Pomalidomide (CC-407) was donated by Celgene Corporation (Summit, NJ, USA) and was dissolved in dimethylsulfoxide (DMSO) to 1 mg/mL immediately before use. For injection into mice, pomalidomide stock solutions were diluted in sterile 0.9% (v/v) normal saline. The final concentration of DMSO in all experiments was <0.01% (v/v).

### Generation of BM-Derived DCs

BALB/c BM-derived immature DCs (imDCs) were generated as described previously (15, 16, 34). Briefly, BM was harvested from the femurs and tibiae of mice and cultured in RPMI-1640 medium (Gibco-BRL) supplemented with 10% (v/v) FBS (Gibco-BRL) and 1% (w/v) PS in the presence of 20 ng/mL recombinant murine (rm) granulocyte macrophage-colony stimulating factor (R&D Systems, Minneapolis, MN, USA) and 10 ng/mL recombinant mouse interleukin 4 (rmIL-4; R&D Systems). On culture days 2 and 4, half of the medium was removed and replaced with fresh medium containing cytokines. On day 6, imDCs were purified by positive selection with CD11c^+^-magnetic beads (Miltenyi Biotec Inc., Auburn, CA, USA). Mature DCs were generated by further cultivation, for 48 h, of CD11c^+^ DCs with 10 ng/mL recombinant murine tumor necrosis factor-alpha (rmTNF-α; R&D Systems), 10 ng/mL rmIL-1β (R&D Systems), and 10 ng/mL rmGM-CSF (R&D Systems).

### Generation of Dying Myeloma Cell-Loaded DCs

The generation of dying myeloma cell-loaded DCs was performed as described previously (15, 34). Briefly, MOPC-315 tumor cell death was induced by gamma irradiation (100 Gy, Gammacell-1000 Elite; MDS Nordion, Ottawa, ON, Canada) followed by overnight culture in FBS-free RPMI-1640. The cells were mixed with imDCs 2 h after maturation in a 2:1 ratio (DCs: dying tumor cells).

### Animal Vaccination

The following four vaccination groups (five mice per group) were established: (1) PBS control, (2) DC vaccination, (3) pomalidomide + dexamethasone, and (4) DC vaccination + pomalidomide + dexamethasone. On day 0, mice were injected subcutaneously with 5 × 10^5^ MOPC-315 cells in a volume of 0.1 mL into the right flank. After tumor growth, pomalidomide (0.06 mg/kg/day) was orally administered once a day for 25 days with a 3-day break after the first 11-day dosing period. Each dose of DCs (1 × 10^6^ per mouse) was injected subcutaneously into the left flank of BALB/c mice in a volume of 0.1 mL PBS on days 14, 18, 28, and 32, and dexamethasone (0.6 mg/kg/day) was injected intravenously in a volume of 0.1 mL on days 10, 17, 24, and 31. To assess the antitumor status of vaccinated mice, the length, width, and height of each tumor were measured every 3–4 days using a Vernier caliper, and the tumor volume was calculated using the standard formula for the volume of an ellipsoid: *V* = 4/3π(length × width × height/8). To assess the survival prolongation of vaccinated mice, the mice were euthanized when the tumor reached 1,000 mm^3^, which was considered as a death due to the size of tumor.

### Phenotypic Analysis of Splenocytes From Vaccinated Mice

The splenocytes of vaccinated mice were isolated 7 days after the final DC vaccination (day 39), and the splenocyte phenotypes were characterized by their cell surface markers using fluorescently labeled monoclonal antibodies (mAbs) and analyzed using flow cytometry. The cells were stained with the following mAbs (all from eBioscience, San Diego, CA, USA): CD11b-FITC, CD11b-PE, Gr-1-PE, CD4-APC, CD4-PE, CD8-FITC, CD49b-PE, CD44-PE, CD62L-FITC, CD69-FITC, CD25-FITC, Foxp3-APC, F4/80-FITC, and CD206-APC. Isotype-matched controls were run in parallel. Cell debris was eliminated by forward- and side-scatter gating. The samples were acquired on a FACS Calibur cell sorter (Becton Dickinson, Mountain View, CA, USA) and the data were analyzed using WinMDI ver. 2.9 software (Biology Software Net: http://en.bio-soft.net/other/WinMDI.html).

### T Cells and NK Cells Activities in Vaccinated Mice

The T cells and NK cells activities were investigated as described previously (14–16, 34). Briefly, splenocytes (1 × 10^6^) isolated from vaccinated mice 7 days after the final DC vaccination (day 39) were added to 24-well plates and restimulated with irradiated MOPC-315 cells (5 × 10^5^ cells) for 5 days in RPMI-1640 (Gibco-BRL) containing 10% FBS (Gibco-BRL) and 1% PS supplemented with 20 ng/mL rmIL-2 (R&D Systems). After restimulation, the splenocytes were assessed for T lymphocytes and NK cells activities using a mouse IFN-γ enzyme-linked immunospot (ELISPOT) assay (BD Bioscience). The MOPC-315 cell line and NK-sensitive YAC-1 cell line were used as target cells, and the spots were enumerated by using an ImmunoSpot Image Analyzer system (Cellular Technology Limited, Shaker Heights, Cleveland, OH, USA) for automated plate scanning, imaging, and spot counting.

### *In Vitro* Analysis of Cytokine Production from Vaccinated Mice

The splenocytes and tumor from vaccinated mice were isolated 7 days after the final DC vaccination (day 39), and the cytokine [interferon-gamma (IFN-γ), IL-10, and VEGF] production from vaccinated mice was determined using the BD OptEIA™ enzyme-linked immunosorbent assay (ELISA) (BD Bioscience). Supernatants from cultures of restimulated splenocytes and from tumor of all vaccinated mice were assayed to measure the production of Th1- and regulatory-polarizing cytokines. Each sample was analyzed in triplicate, and the mean absorbance for each set of standards and samples was calculated.

### Intracellular Staining Assay of Tregs and Macrophages Generated in the Spleens of Vaccinated Mice

To evaluate the proportion of Tregs and macrophages, the splenocytes of vaccinated mice were isolated 7 days after the final DC vaccination (day 39), and 1 × 10^6^ splenocytes from vaccinated mice were harvested, washed, and stained with surface-staining antibodies for Tregs (CD4-PE and CD25-FITC) and macrophages (CD11b-FITC and F4/80-PE) for 30 min at 4°C. Fc block was added before incubation with surface antibodies. The cells were washed and permeabilized with FACSTM Permeabilizing Solution 2 (BD Bioscience) for 30 min at room temperature. After washing twice, the cells were stained with an intracellular staining antibody for Tregs (Alexa Fluor-conjugated Foxp3 antibody; Miltenyi Biotec, Bergisch Gladbach, Germany) and macrophages (CD206-APC) for 30 min at room temperature. The samples were acquired on a FACS Calibur cell sorter (Becton Dickinson) and the data were analyzed using WinMDI Ver. 2.9 software.

### Statistical Analyses

GraphPad Prism 4 software (GraphPad, San Diego, CA, USA) was used to analyze tumor growth and to determine statistical significance of difference between groups by applying a one-way ANOVA. Survival of the vaccinated mice was analyzed using SigmaPlot 10.0 (Systat software Inc., San Jose, CA, USA). A *P* value <0.05 was considered significant. Means ± SDs are shown.

## Results

### DC Vaccination in Combination With Pomalidomide Induces Synergistic Antimyeloma Immunity Effect

**Figure 1:**
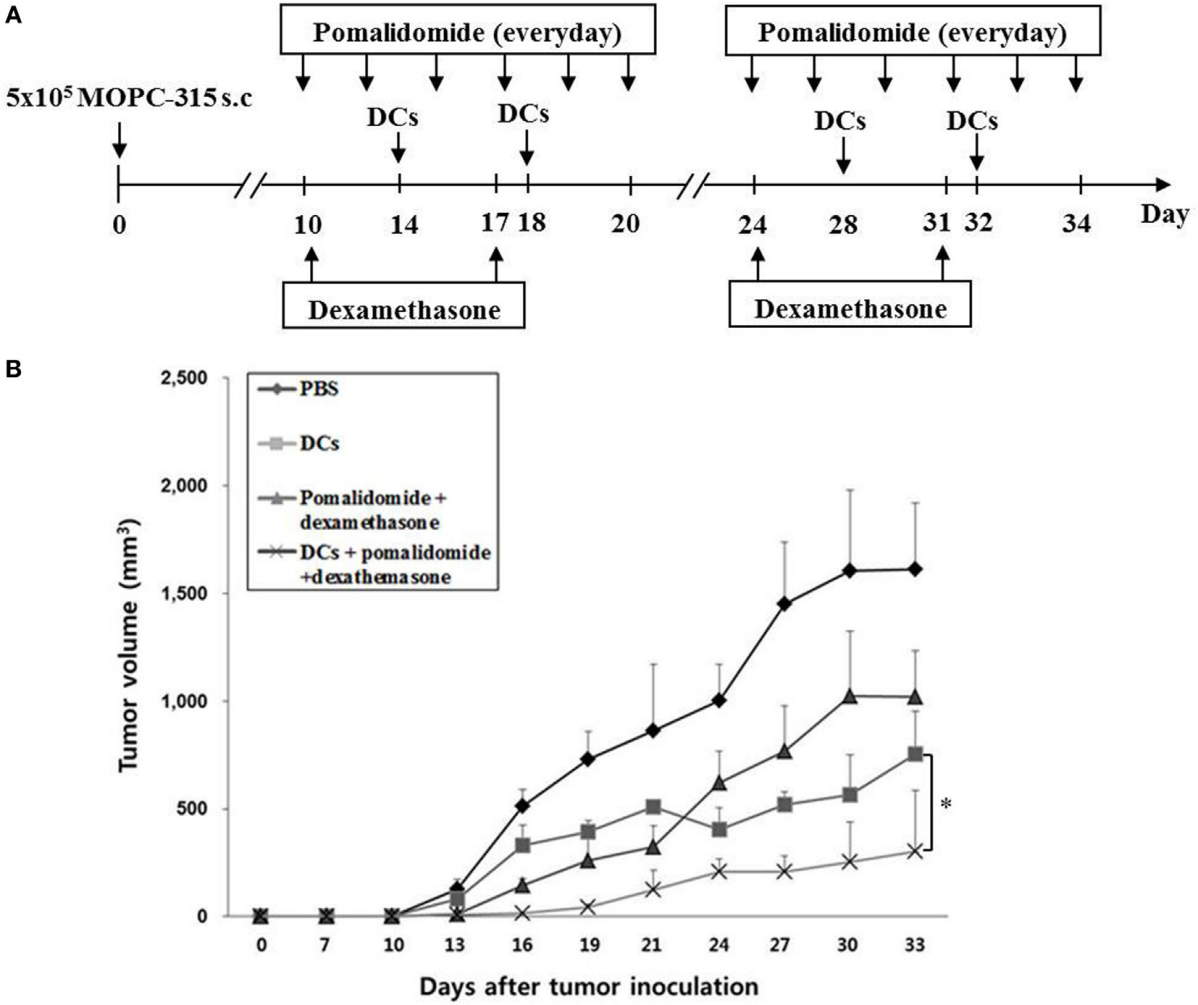
*In vivo* animal vaccination. Four vaccination groups were established: (1) PBS control, (2) dying myeloma cells-loaded dendritic cell (DC) vaccination, (3) pomalidomide + dexamethasone, and (4) DC vaccination + pomalidomide + dexamethasone. **(A)** Schematic representation of the combination of dendritic cells (DCs) + pomalidomide + dexamethasone. On day 0, MOPC-315 cells (5 × 10^5^ per mouse) were injected subcutaneously into the right flank of BALB/c mice. After tumor growth, pomalidomide (0.06 mg/kg/day) was orally administered once a day for 25 days with a 3-day break after the first 11-day dosing period. Each dose of DCs (1 × 10^6^ per mouse) was injected subcutaneously into the left flank of each BALB/c mouse in a volume of 0.1 mL PBS on days 11, 15, 25, and 29, and dexamethasone (0.6 mg/kg/day) was injected intravenously on days 10, 17, 24, and 3. **(B)** The data are means ± SEs and are representative of two independent experiments. The combination of DCs + pomalidomide + dexamethasone significantly inhibited tumor growth (*P < 0.05 on day 27) and induced a long-term systemic antimyeloma immune response (30 days).

Our previous study demonstrated that murine DCs maturated with GM-CSF, TNF-α, and IL-1β expressed higher levels of several molecules related to DC maturation and produced higher levels of IL-12p70 and lower levels of IL-10 compared to imDCs (15). In this study, to evaluate the antitumor efficacy of pomalidomide alone or in combination with dexamethasone in the treatment of myeloma, we established a mouse model of myeloma. After tumor growth, pomalidomide (0.06 mg/kg/day) was orally administered once a day for 25 days with a 3-day break after the first 11-day dosing period, and dexamethasone (0.6 mg/kg/day) was injected intravenously on days 10, 17, 24, and 31 (Figure 1A).

Treatment with pomalidomide plus dexamethasone, which is a commonly used clinical treatment regimen, dramatically inhibited tumor growth compared with the PBS control or pomalidomide alone in the myeloma mouse model (Figures S1A,B in Supplementary Material). Therefore, combination therapy of DCs with pomalidomide and dexamethasone was further in the mouse model (Figure 1A). All tumor-bearing mice vaccinated with PBS showed rapid tumor growth that led to their death within 3 weeks. In contrast, tumor-bearing mice vaccinated with DCs, pomalidomide + dexamethasone, and DCs + pomalidomide + dexamethasone displayed significantly inhibited tumor growth compared with the PBS control group. Treatment with a combination of DC vaccination + pomalidomide + dexamethasone achieved a greater inhibition of tumor growth (*P* < 0.05) compared to pomalidomide + dexamethasone or DCs alone (Figure 1B; Figure S2A in Supplementary Material). Survival in mice that received a combination of DCs + pomalidomide + dexamethasone was prolonged compared to that of mice that received the pomalidomide + dexamethasone, or DCs alone (Figure S2B in Supplementary Material). These results indicated that DCs + pomalidomide + dexamethasone induced a long-term systemic antimyeloma effect in the mouse myeloma model.

### Activation of T Lymphocytes and NK Cells by DC Vaccination Plus Pomalidomide and Dexamethasone

**Figure 2:**
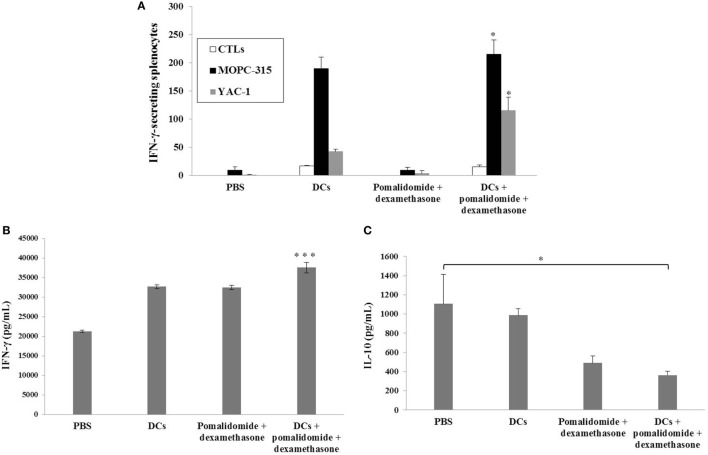
Activation of T lymphocytes and natural killer (NK) cells by vaccination with dendritic cells (DCs) plus pomalidomide and dexamethasone. **(A)** The number of IFN-γ secreting lymphocytes in spleens of mice treated with PBS, DCs, pomalidomide + dexamethasone, and DCs + pomalidomide + dexamethasone were counted using the IFN-γ enzyme-linked immunospot assay. DC vaccination combined with pomalidomide and dexamethasone injection significantly increased the number of IFN-γ-secreting lymphocytes targeting MOPC-315 and YAC-1 cells compared with the other groups. Indicating the tumor-inhibitory effects of DCs + pomalidomide + dexamethasone resulted from the T lymphocyte- and NK cell-mediated activities (*P < 0.05). **(B)** IFN-γ and **(C)** IL-10 production from the splenocytes of vaccinated mice was evaluated by ELISA. The combination of DCs + pomalidomide + dexamethasone led to production of higher levels of IFN-γ compared to PBS control and DC vaccination (***P < 0.001). In contrast, treatment with pomalidomide + dexamethasone or DCs + pomalidomide + dexamethasone led to production of lower levels of IL-10 compared to PBS control and DC vaccination. DCs + pomalidomide + dexamethasone vaccination showed the lowest levels of IL-10 production among others groups (*P < 0.05). Data shown are means (pg/mL) ± SDs of triplicate cultures from three independent experiments.

**Figure 3:**
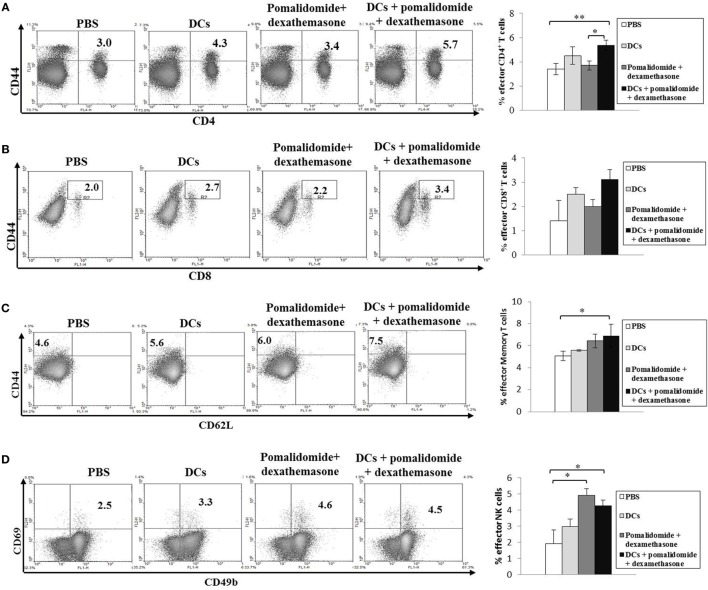
Induction of CD4^+^ T cells, CD8^+^ T cells, memory T cells, and natural killer (NK) cells in the spleens of mice treated with a combination of dendritic cells (DCs) plus pomalidomide and dexamethasone. The proportions of CD4^+^ T cells **(A)**, CD8^+^ T cells **(B)**, memory T cells **(C)**, and NK cells **(D)** were measured by flow cytometry (left panel) and compared by quantitated bar graphs (right panel). Vaccination with DCs + pomalidomide + dexamethasone led to a significant increase of the effector CD4^+^ T compared to the PBS control or pomalidomide + dexamethasone groups (*P < 0.05, **P < 0.012). The proportions of CD8^+^ T cells were highest in the DCs + pomalidomide + dexamethasone group compared to the other groups. Significant increases of effector memory T cells, and effector NK cells were evident in the DCs + pomalidomide + dexamethasone combination group compared with the PBS control group (*P < 0.05). The data are representative of three independent experiments.

To evaluate the activation of T lymphocytes and NK cells to DC vaccination *in vivo* myeloma model, splenocytes from each group of vaccinated mice were prepared for IFN-γ ELISPOT assays. MOPC-315 and YAC-1 cells were used as the target cells. In comparison with the PBS control, treatment with pomalidomide + dexamethasone did not increase the number of IFN-γ-secreting splenocytes against MOPC-315 cells, whereas vaccination with DCs + pomalidomide + dexamethasone led to a significant increase in IFN-γ-secreting splenocytes against MOPC-315 cells compared to the other groups (*P* < 0.05). Furthermore, vaccination with DCs + pomalidomide + dexamethasone significantly increased the cytotoxicity of NK cells as evident by the number of IFN-γ-secreting splenocytes against YAC-1 cells compared to PBS control, DCs alone, and pomalidomide + dexamethasone (*P* < 0.05; Figure 2A). These results indicated that the tumor-inhibitory effects of DCs + pomalidomide + dexamethasone resulted from T lymphocyte- and NK cell-mediated cytotoxicity responses. In this study, vaccination with DCs + pomalidomide + dexamethasone led to the production of higher levels of IFN-γ compared to the PBS control, DCs alone, or pomalidomide + dexamethasone (Figure 2B). In contrast, IL-10 production by DCs + pomalidomide + dexamethasone was lower compared to the PBS control, DCs alone, or pomalidomide + dexamethasone (Figure 2C). These results suggested that the combination of DCs + pomalidomide + dexamethasone enhanced Th1 immune responses in addition to T lymphocytes and NK cells responses. Regarding the induction of effector cells, DCs + pomalidomide + dexamethasone showed significantly increased percentages of effector CD4^+^ T cells (Figure 3A), effector CD8^+^ T cells (Figure 3B), effector memory T cells (Figure 3C), effector NK cells (Figure 3D), and M1 macrophages (Figures 5A,B) compared with the other groups.

### Efficient Inhibition of Myeloid-Derived Suppressor Cells (MDSCs), M2 Macrophages, and Regulatory T Cells (Tregs) With DC Vaccination Plus Pomalidomide and Dexamethasone

**Figure 4:**
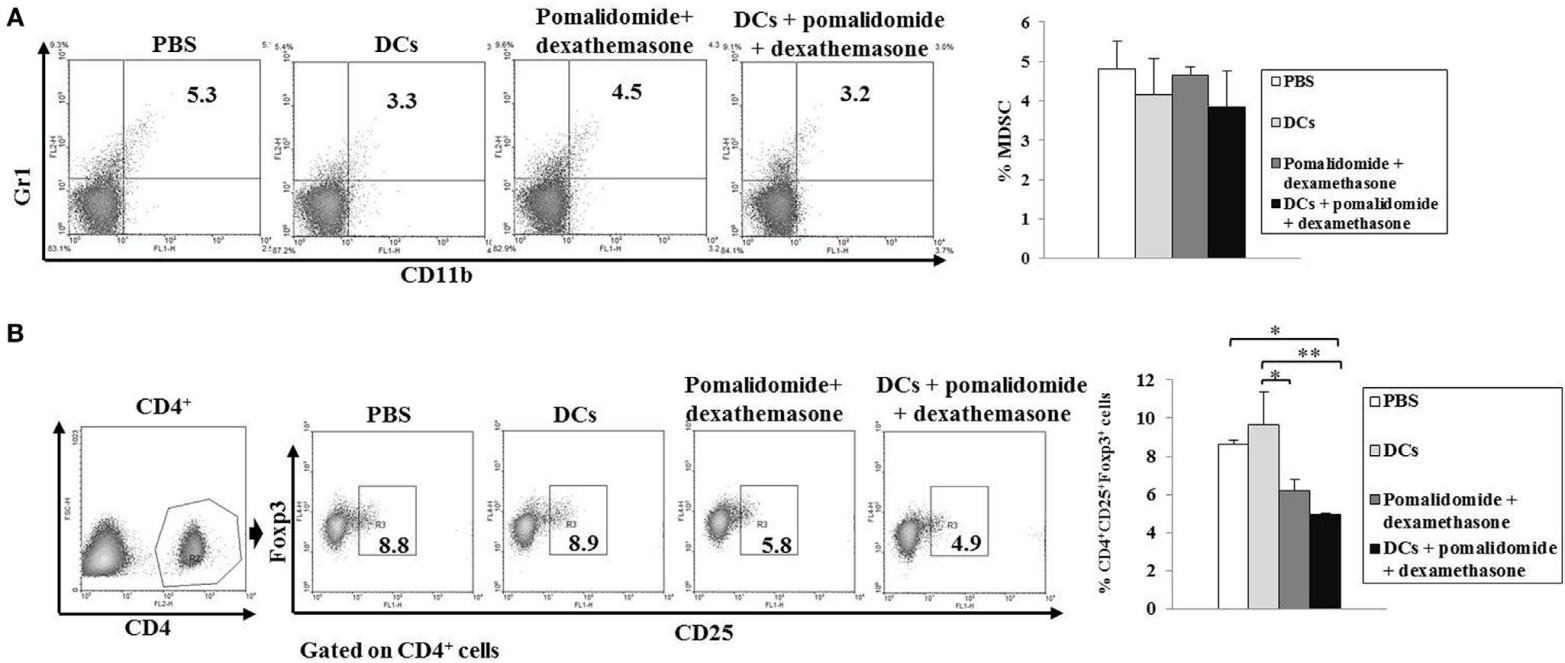
Inhibition of myeloid-derived suppressor cells (MDSCs) and Tregs in the spleens of mice treated with dendritic cells (DCs) plus pomalidomide and dexamethasone. The proportions of MDSCs (CD11b^+^Gr-1^+^) cells **(A)** and CD4^+^CD25^+^Foxp3^+^ Treg cells **(B)** were measured by flow cytometry (left panel) and compared by quantitative bar graphs (right panel). Vaccination with DCs + pomalidomide + dexamethasone led to decreased generation of splenic MDSCs compared to the PBS control, DCs alone, or pomalidomide + dexamethasone. The proportions of Tregs were significantly increased in the PBS control and DC vaccination group compared with the groups injected with pomalidomide + dexamethasone after tumor inoculation. The DCs + pomalidomide + dexamethasone combination group showed the lowest proportions of splenic Tregs compared to the other groups (*P < 0.05, **P < 0.012). Data are representative of more than three experiments.

**Figure 5:**
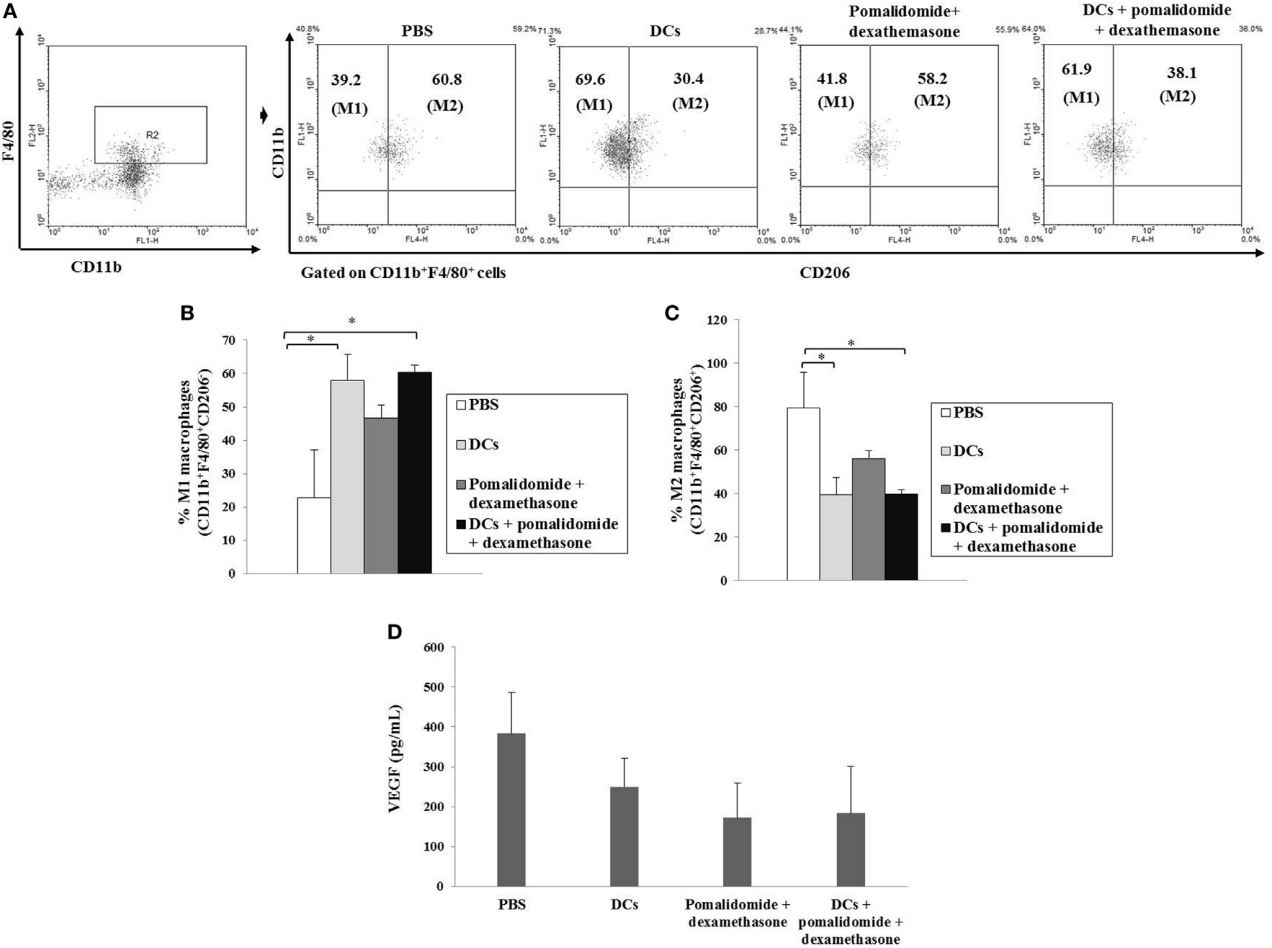
Enhanced M1 and impaired M2 macrophages polarization and reduced angiogenesis cytokine production vial vaccination with dendritic cells (DCs) plus pomalidomide and dexamethasone. **(A)** The proportions of M1 macrophages (CD11b^+^F4/80^+^CD206^−^) and M2 macrophages (CD11b^+^F4/80^+^CD206^+^) in the spleens of vaccinated tumor-bearing mice were gated on CD11b^+^F4/80^+^ cells using flow cytometry. **(A,B)** The proportions of M1 macrophages were significantly increased in the DCs and DCs + pomalidomide + dexamethasone vaccination groups compared with the PBS control group (*P < 0.05). **(A,C)** The proportions of M2 macrophages were significantly decreased in the DCs and DCs + pomalidomide + dexamethasone vaccination groups compared with the PBS control group (*P < 0.05). The data are representative of three independent experiments. The production of angiogenesis cytokine vascular endothelial growth factor (VEGF) **(D)** in the tumor of tumor-bearing mice was evaluated by ELISA. Treatment with pomalidomide + dexamethasone groups led to production of the lowest level of VEGF compared to DCs alone, or PBS control groups. Data are representative of more than three experiments.

To explore the immunological mechanisms underlying the enhanced tumor-specific immune response shown above, we evaluated the effects of combination therapy on the proportions of MDSCs (CD11b^+^Gr1^+^), M2 macrophages (CD11b^+^F4/80^+^CD206^+^ cells), and Tregs (CD4^+^CD25^+^FoxP3^+^ cells). Vaccination with DCs + pomalidomide + dexamethasone decreased the generation of splenic MDSCs compared to the PBS control, DCs alone, or pomalidomide + dexamethasone (Figure 4A). While the proportions of Tregs were significantly increased in the PBS control and DC vaccination alone compared with the groups injected with pomalidomide + dexamethasone in the mouse myeloma model. Interestingly, the combination of DCs + pomalidomide + dexamethasone exhibited the lowest proportion of splenic Tregs (*P* < 0.05; Figure 4B). Moreover, treatment with DCs + pomalidomide + dexamethasone produced a significant decrease in the proportion of splenic M2 macrophages compared to the PBS control (Figures 5A,C). These findings suggested that the DCs + pomalidomide + dexamethasone enhanced the therapeutic antitumor immunity by inhibiting the immunosuppressive cells in the tumor microenvironment during the vaccination phases.

### Efficient Suppression of Angiogenesis Cytokine Production by DC Vaccination Plus Pomalidomide and Dexamethasone in Tumor Microenvironment of Myeloma-Bearing Mice

To investigate the immunological mechanisms underlying the observed enhancement of the tumor-specific immune response, we evaluated the effects of combination therapy with DC vaccination + pomalidomide + dexamethasone on the production of angiogenesis cytokine VEGF. Compared to the treatment groups, the PBS control displayed the highest level of VEGF. In contrast, treatment with pomalidomide + dexamethasone led to the production of the lowest level of VEGF compared to DCs alone or the PBS control groups (Figure 5D), suggesting that the combination therapy with DC vaccination + pomalidomide + dexamethasone suppressed angiogenesis by inhibiting VEGF production in the tumor microenvironment of myeloma-bearing mice.

## Discussion

The fundamental goal of immunotherapy in the treatment of MM is to boost tumor-specific immunity and eliminate malignant cells. Based on *in vitro* and preclinical studies, DC-based tumor vaccines have the potential to induce antimyeloma immunity mediated by CTLs (11, 35). However, eliciting a clinically meaningful antitumor effect is challenging and the results remain inconclusive, with validation required in larger prospective randomized controlled studies (36).

The tumor microenvironment is essential for MM growth, progression, and drug resistance by virtue of survival signals and secretion of growth and proangiogenic factors that inhibit tumor-specific T cells and hamper the efficacy of DC vaccination (37, 38). The development of therapeutic strategies to manipulate the tumor microenvironment is considered a major obstacle to curing this disease. In previous studies, we observed that antimyeloma immunity was synergistically elicited by the combination therapy of DCs with lenalidomide as an immunomodulatory adjuvant (10, 22). In the present study, we examined the synergistic effect of DC vaccination plus pomalidomide and dexamethasone for treating myeloma in a mouse model of myeloma. A previous clinical report demonstrated that pomalidomide + low-dose dexamethasone can significantly improve progression-free survival in patients with relapsed and refractory MM (25). Furthermore, in a preclinical study (26), pomalidomide displayed significant therapeutic activity against central nervous system lymphoma with a major impact on the tumor microenvironment with an increase in M1 macrophages and NK cells. Consistently, in this study, the group vaccinated with DCs + pomalidomide + dexamethasone displayed decreased tumor growth, prolonged survival, induced NK cell, and T lymphocytes responses associated with strong antimyeloma activities against myeloma cells and NK-sensitive YAC-1 cells, increased numbers of effector cells (such as CD4^+^ T cells, CD8^+^ T cells, memory T cells, NK cells, and M1 macrophages), and decreased numbers of suppressor cells including MDSCs, Tregs, and M2 macrophages in the spleens of the vaccinated mice. Tregs, MDSC, and M2 macrophages are important in the potent immunosuppression that is mediated by tumor secreting cytokines. The inhibition of Tregs, MDSC, and M2 macrophage accumulation in spleen may enhance systemic cell-mediated immunity through the activation of DCs or CTLs.

Our previous study (39) demonstrated that lenalidomide enhances the function of DCs generated from patients with MM by inhibiting the generation of immunosuppressive cells, inducing naïve T cells toward Th1 polarization, and generating potent myeloma-specific CTLs. In this recent study, DCs combined with pomalidomide and dexamethasone induced the activation of cell-mediated immunity by increasing Th1-specific immune responses, as evidenced by high-level production of IFN-γ, and decreased regulatory-specific immune response that was evident as the low-level production of IL-10 and VEGF. The collective observations are evidence of a systemic immune response with the potential to treat a myeloma.

Mouse models of myeloma are known to play a critical tool for studying the mechanisms of disease resistance, pathogenesis, and the development of new therapeutics against malignancies (35, 40–42). This study has some limitation to interpret data due to subcutaneous injection of MOPC-315 cells for making plasmacytoma rather than BM involvement model for myeloma.

In conclusion, the present results suggest that pomalidomide plus dexamethasone are potent immune adjuvants in combination with DC vaccination in a murine myeloma model. In comparison with the PBS control, DC vaccination alone, and pomalidomide plus dexamethasone, the combination of DCs + pomalidomide + dexamethasone led to the synergistic enhancement of antimyeloma activity by inhibiting the generation of immune-suppressive cells and regulatory immune response as well as enhancing the effector cells and Th1 immune response during myeloma progression. For this reason, we suggest that an appropriate multimodal treatment using a DC vaccine combined with IMiDs. Since the combination of DCs + pomalidomide + dexamethasone inhibits MM growth, we are considering to further improve the effectiveness of this combination. The test on tumor-free mice is needed to clarify the mechanisms underlying the activity of this combination, and understand the important role of each factor in the outcome of this combination modality.

## Ethics Statement

All animal care, experiments, and euthanasia were performed in accordance with protocols approved by the Chonnam National University Animal Research Committee.

## Author Contributions

M-CV, S-HJ, and J-JL designed the study. M-CV, SY, T-HC, H-JL, H-SP, and TL performed the research and analyzed the data. M-CV and J-JL wrote the article. J-JL, S-HJ, and H-JK contributed intellectually to the research.

## Conflict of Interest Statement

The authors declare that the research was conducted in the absence of any commercial or financial relationships that could be construed as a potential conflict of interest.

## Supplementary Material

Figure S1 Antitumor efficacy of pomalidomide alone or in combination with dexamethasone in the mouse myeloma model. **(A,B)** Representative pictures of mice vaccinated with pomalidomide + dexamethasone showing dramatic inhibition of tumor growth compared with the PBS control or pomalidomide alone. Experiments consisted of five mice per group.

Figure S2 **(A)** Representative pictures of mice vaccinated with DCs + pomalidomide + dexamethasone showing significant inhibition of tumor growth compared to the PBS control, DC vaccination, and pomalidomide plus dexamethasone (**P* < 0.05 on day 27). **(B)** DCs + pomalidomide + dexamethasone vaccination induced a long-term systemic antimyeloma immune response (30 days) compared to the PBS control, DC vaccination, and pomalidomide plus dexamethasone. Experiments consisted of five mice per group.
