# Supplementary figures and images for: Synergistic Antimyeloma Activity of Dendritic Cells and Pomalidomide in a Murine Myeloma Model
Source: Front Immunol. 2018 Aug 3;9:1798. doi: 10.3389/fimmu.2018.01798 (PMC6085413; doi:10.3389/fimmu.2018.01798)

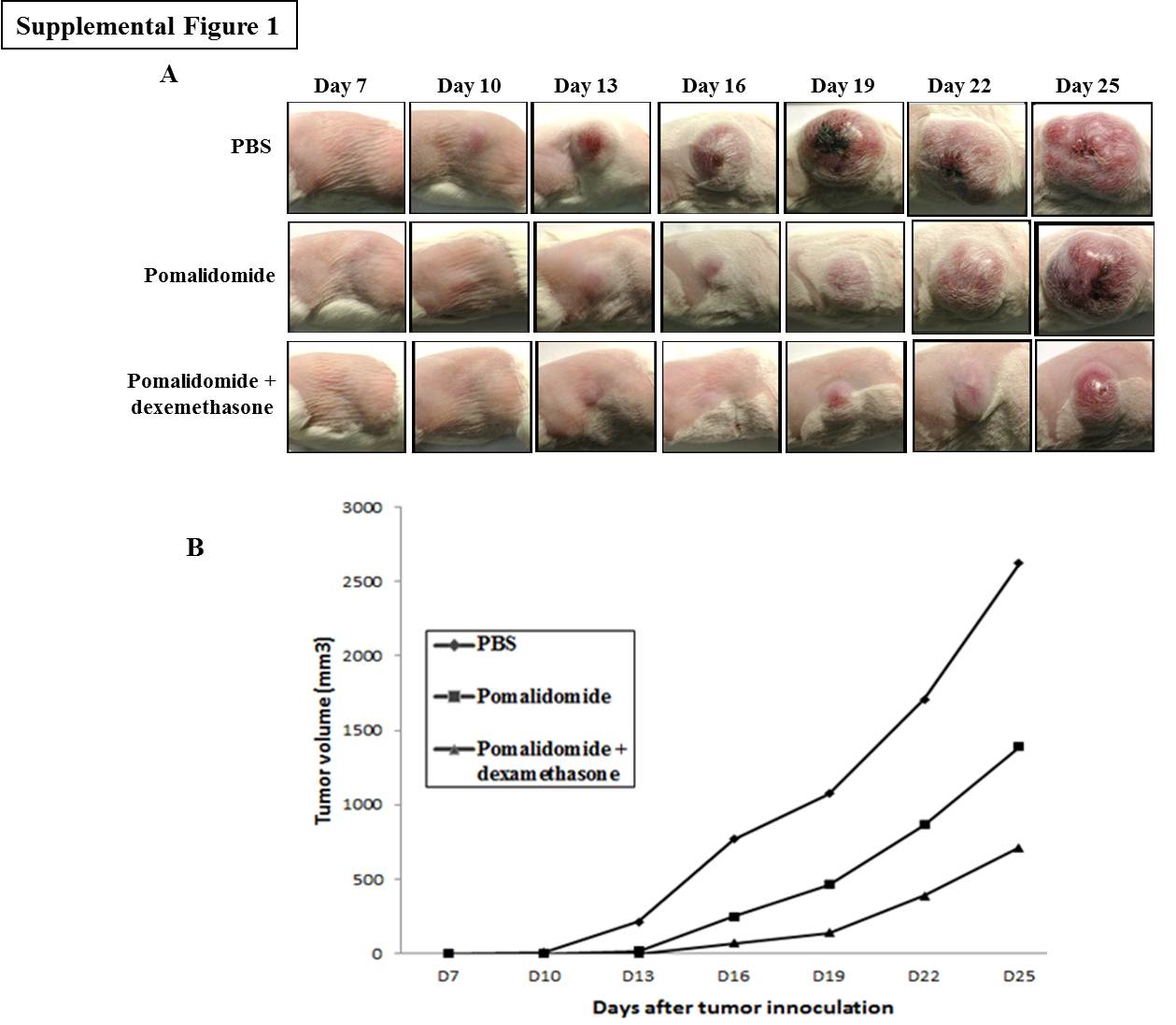

Supplement: Figure S1 — Antitumor efficacy of pomalidomide alone or in combination with dexamethasone in the mouse myeloma model. (A,B) Representative pictures of mice vaccinated with pomalidomide + dexamethasone showing dramatic inhibition of tumor growth compared with the PBS control or pomalidomide alone. Experiments consisted of five mice per group. [file Image_1.jpeg]

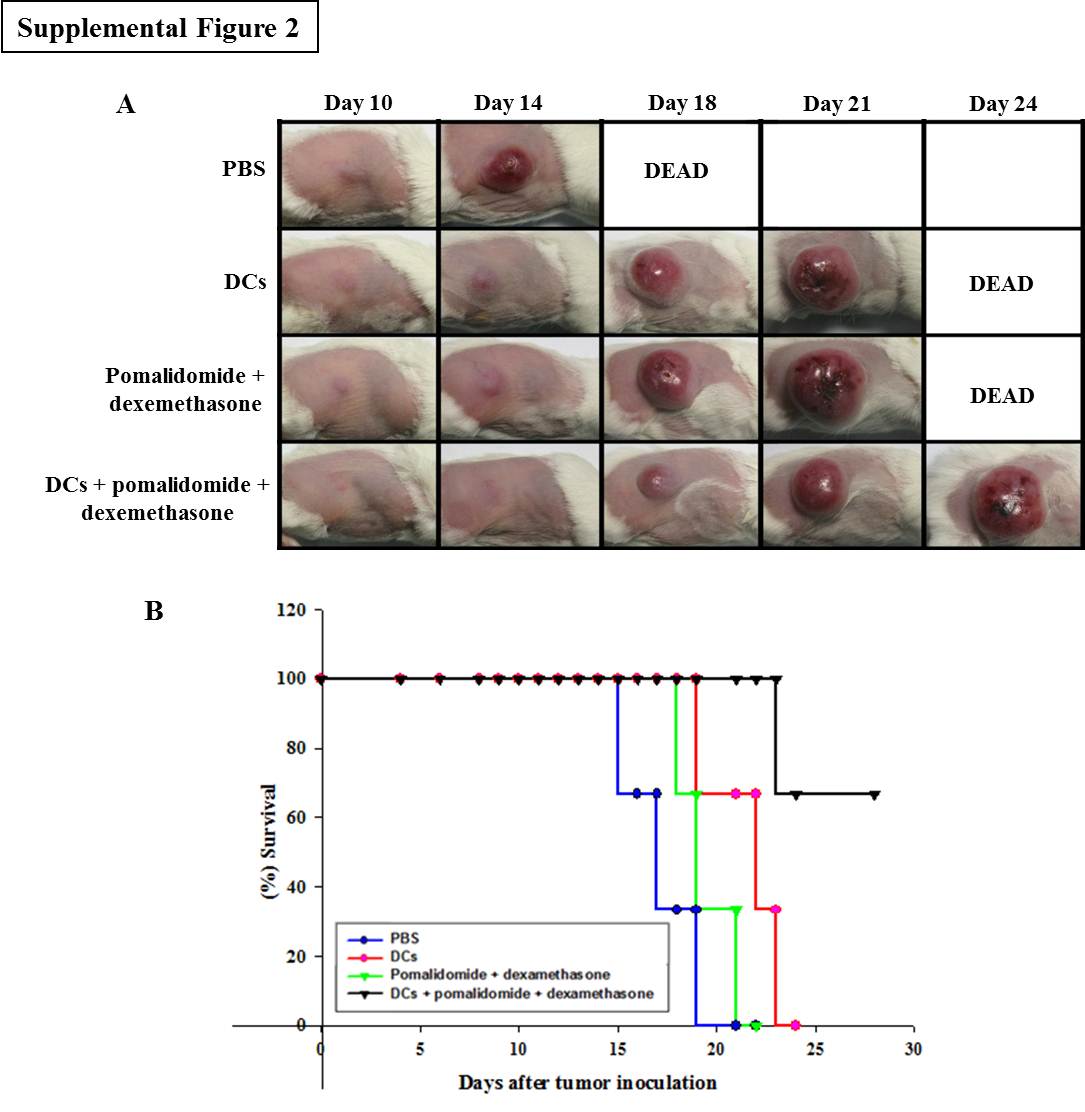

Supplement: Figure S2 — (A) Representative pictures of mice vaccinated with DCs + pomalidomide + dexamethasone showing significant inhibition of tumor growth compared to the PBS control, DC vaccination, and pomalidomide plus dexamethasone (*P < 0.05 on day 27). (B) DCs + pomalidomide + dexamethasone vaccination induced a long-term systemic antimyeloma immune response (30 days) compared to the PBS control, DC vaccination, and pomalidomide plus dexamethasone. Experiments consisted of five mice per group. [file Image_2.jpeg]
